# Evaluation of interproximal reduction in individual teeth, and full arch assessment in clear aligner therapy: digital planning versus 3D model analysis after reduction

**DOI:** 10.1186/s40510-022-00403-w

**Published:** 2022-03-07

**Authors:** Amirtha Hariharan, Sarah Abu Arqub, Vaibhav Gandhi, Lucas Da Cunha Godoy, Chia-Ling Kuo, Flavio Uribe

**Affiliations:** 1 Division of Orthodontics, Department of Craniofacial Sciences, University of Connecticut Health, 263 Farmington Ave, Farmington, CT 06032 USA; 2 Division of Orthodontics, University of Louisville, Louisville, KY USA; 3 Connecticut Convergence Institute for Translation in Regenerative Engineering, University of Connecticut Health, Farmington, CT USA

**Keywords:** Interproximal reduction, Invisalign®, ClinCheck®, 3D simulation

## Abstract

**Aim:**

To evaluate the correspondence between the interproximal reduction (IPR) performed clinically and that programmed in ClinCheck® and further assess which teeth showed an amount of implemented IPR (I-IPR) that corresponds with that programmed in ClinCheck®.

**Materials and methods:**

Pre- (T0) and post-treatment (T1) ClinCheck® digital models for 75 subjects (30 males and 45 females), mean age (38 ± 15) years, were included. To calculate the amount of I-IPR, Ortho Analyzer software (3Shape, Copenhagen, Denmark) was used to measure the mesiodistal widths for the maxillary and mandibular teeth from second premolar to the contralateral second premolar on the initial (T0) and final (T1) STL models. I- IPR performed by tooth was obtained by comparing the mesiodistal width of each tooth at T0 and T1. The amount of programmed IPR (P-IPR) in ClinCheck® was compared to that implemented clinically using the following formula: IPR difference = (P-IPR) − (I-IPR).

**Results:**

Statistically significant differences were observed between the average value of digitally programmed and implemented IPR per tooth for both the maxillary (*p* < .0001) and mandibular (*p* < .0001) teeth. The mean P-IPR for the maxillary teeth was 0.28 ± 0.16 mm versus the mean I-IPR of 0.15 ± 0.15 mm. In the mandibular arch, the mean P-IPR was 0.31 ± 0.17 mm, while the I-IPR was 0.17 ± 0.16 mm. The mean I-IPR was consistently lower than the mean P-IPR regardless of teeth and sites (*p* < 0.0001). The difference between the P-IPR compared to the I-IPR was larger for mandibular anterior teeth than for maxillary anterior teeth (*p* = 0.0302) and larger for maxillary posterior teeth than mandibular posterior teeth (*p* = 0.0059).

**Conclusion:**

The amount of implemented-IPR in clear aligner therapy is less than that digitally programmed for most teeth. Regardless of the regions, I-IPR was consistently lower than that programmed. Mandibular anterior teeth and maxillary posterior teeth showed greater discrepancy between P-IPR and I-IPR than the maxillary anterior and mandibular posteriors. Further prospective studies are needed to determine the factors affecting the precision of IPR and the clinical implications of a significantly reduced I-IPR on treatment outcomes.

## Introduction

The advantages offered by clear aligners over traditional fixed appliances related to improved esthetics, comfort, and fewer emergencies[1] have led to the increased popularity and demand on clear aligner therapy, specifically the Invisalign® system (Align Technology, San Jose, California). This system relies primarily on enamel interproximal reduction (IPR) as a space gaining procedure [2], mainly for crowded dentitions which constitutes the highest percentage of cases treated with clear aligners [3]. On the other hand, with the evolution of Invisalign® system, virtual simulation of teeth movement became possible through the ClinCheck® (Align Technology, San Jose, California) [4]. Specifically, this software allows a virtual plan capable of displaying the amount of planned IPR, location of resin attachments, and specific tooth movements simulated on 3D models prior to fabrication of the clear aligners.

IPR is an adjunct clinical procedure involving the reduction, anatomic recontouring, and protection of proximal enamel surfaces of permanent teeth [5].

The procedure of IPR entails approximately 0.3–0.5 mm removal of the outer enamel on the interproximal surfaces of teeth [6]. It mainly allows gaining space to relief crowding and facilitate tooth movement and alignment when extraction is undesirable [7]. Other claimed advantages include reduction in treatment time [6], providing greater contact point areas therefore greater stability [8] and reduction of open gingival embrasures (black triangles) [7]. IPR can further assist in reducing Bolton’s disharmonies [9] and achieving treatment objectives without compromising the integrity of periodontal and dental tissues [10].

Despite a lack of evidence that abraded enamel might be more susceptible to dental caries [11], the acceptance of IPR as a mean to provide space is still hindered by the notion and there is no doubt that maintenance of an adequate enamel layer thickness is crucial to prevent the rapid extension of decay to dentin. Furthermore, careful performance of IPR with proper techniques and exact amount of enamel needed are a prerequisite to avoid potential harms to dental and gingival structures [12].

In the programmed IPR (P-IPR) during ClinCheck® development, the clinician can determine the amount and location of IPR to be performed. Therefore, the amount of enamel to be removed can be quantified according to the clinical case requirements [13]. The amount of IPR performed clinically, implemented IPR (I-IPR), has to correspond to that P-IPR in the ClinCheck® to achieve the desired tooth movements and enable the accuracy of implementing the 3D treatment plan [14]. However, the actual procedure of enamel reduction can be performed using several techniques: discs, strips, or burs, and is largely dependent on clinicians’ skills and comfort [6, 11, 15]. Hence, the accuracy of implementing the P-IPR clinically might be influenced by the various IPR techniques and operators’ skills. Consequently, it is crucial to assess the accuracy of implementing the P-IPR clinically. Few studies have reported on quantitative evaluations of the I-IPR vs P-IPR; two of which found the amount of I-IPR less than that programmed on the ClinCheck® [14, 16]. More recently, Lagana et al. [17] found that the amount of IPR conducted in vivo corresponds to that programmed in ClinCheck®. However, none of these studies assessed the accuracy of P-IPR and I-IPR for each tooth individually, as well as for the entire arch (maxilla or mandible, 2^nd^ premolar to 2^nd^ premolar). Therefore, this retrospective study aimed to evaluate the correspondence and precision between the I-IPR performed clinically and that programmed in the ClinCheck® software (P-IPR). Further, this study aimed to assess which teeth specifically showed an I- IPR that corresponds with the P-IPR. Our null hypothesis was that there is no difference between the amount of implemented and programmed IPR.

## Materials and methods

Approval to conduct this study was obtained from the institutional review board of UCONN Health (20X-048–2). For this retrospective study, ClinCheck® 3D models for 129 subjects who completed or were still undergoing Invisalign® treatment at UCONN Health-Division of Orthodontics were screened for the following inclusion criteria:

- Comprehensive Invisalign® treatment package
- Mild-to-moderate crowding (2–6) mm that did not require extraction
- IPR treatment plan
- Full permanent dentition excluding third molars
- The presence of at least one refinement or final scan following the preliminary simulated treatment plan.

**Fig. 1:**
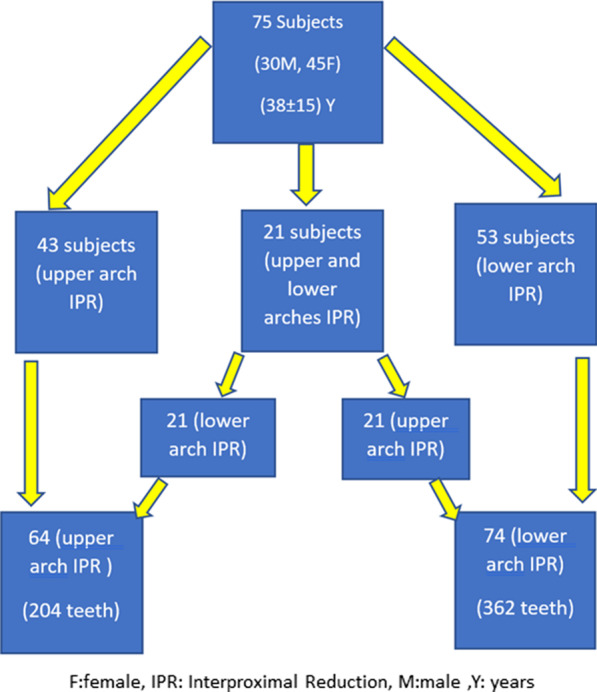
Demographics

Subjects with extractions’ therapy, Invisalign® non-Comprehensive Package therapy, impacted, missing, or supernumerary teeth, and those with prosthetic replacements were excluded. The STL (stereolithographic) models of 75 subjects (30 males and 45 females) were included with the mean age 38 years (± 15), (Fig. 1).

The amount of digitally P-IPR for the maxillary and mandibular arches from second premolar on the right side to the second premolar on the left side were obtained from the initial ClinCheck® setups as planned by each clinician according to the individual patient’s treatment needs and recorded in an Excel spreadsheet. STL files of the initial (T0) and last (T1) dental casts available (final or refinement) were exported from ClinCheck® and imported into Ortho Analyzer software (3Shape, Copenhagen, Denmark). Two trained and calibrated examiners (orthodontic residents), with more than three years’ experience in measuring digital models and using the Ortho Analyzer software, performed the measurements. Additionally, a training session was carried out for them to improve their skills in using the cross-sectional tool of Ortho Analyzer software. According to the manufacturer, the software is calibrated, and its digital caliper could be used with an accuracy of 0.01 mm. Examiner 1 performed all the measurements, and examiner 2 repeated the measurements of 14 digital models, one month after initial measurements, to evaluate reliability of the measurement method (“Appendix”).

**Fig. 2:**
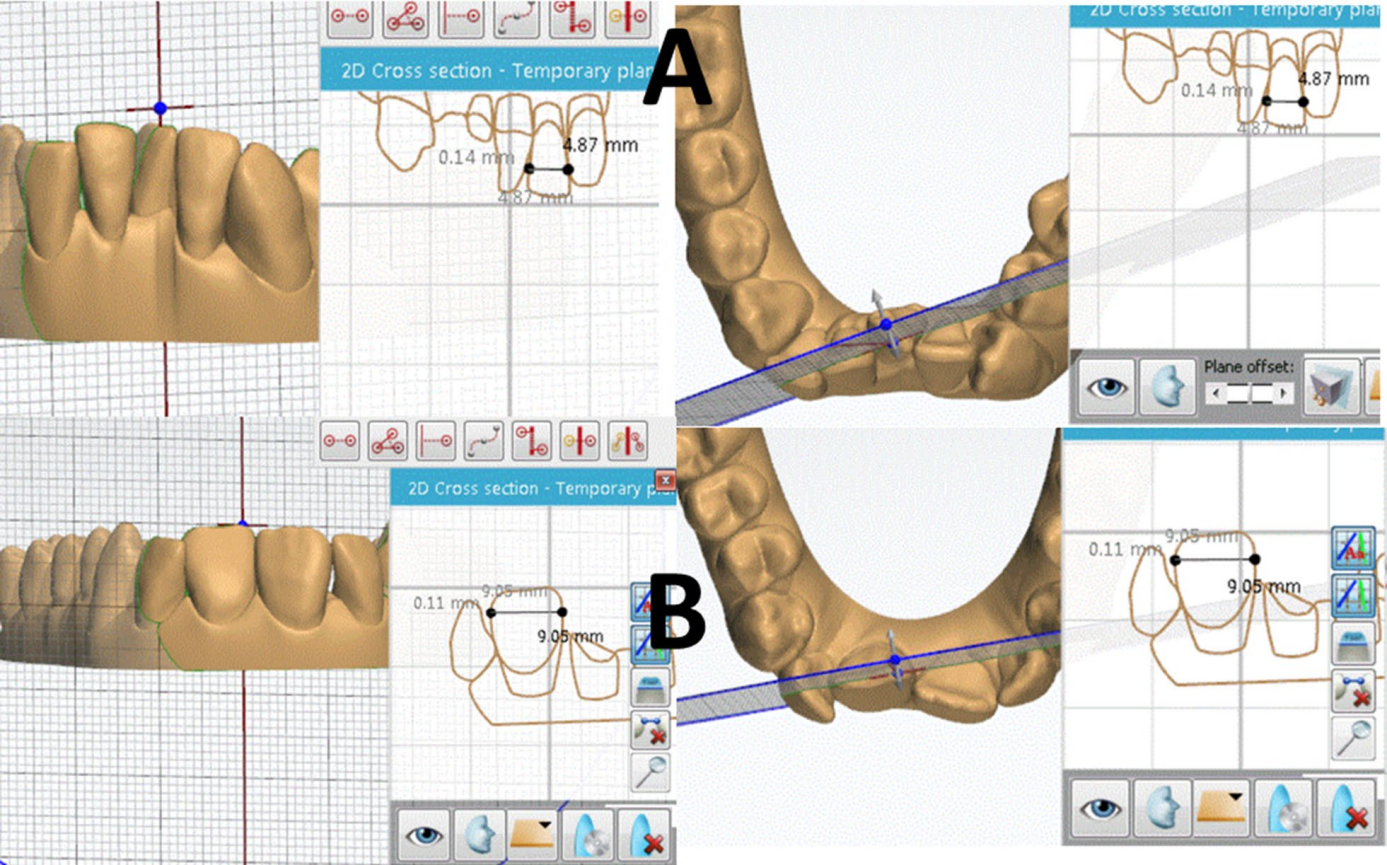
Example for a 2D cross-sectional measurement for mandibular (**A**) and maxillary incisors (**B**)

The mesiodistal widths for the maxillary and mandibular teeth from second premolar to the contralateral second premolar were measured on the initial (T0) and final (T1) STL models for each subject. The models were manipulated in 3D such that each tooth being measured was first oriented perpendicular to the computer screen. Each tooth was 2D sectioned by a line from its mesial to distal height of contour using the cross-sectional tool of the Ortho Analyzer software. Afterward, using the same tool the maximum width of the tooth was measured between the mesial and distal maximum contact point contours in the coronal 2D sectional view (Fig. 2). These procedures allowed more accurate and easier evaluation of the mesiodistal measurements, compared to utilizing different calibers on conventional models (manual or digital), which are primarily dependent on the operator learning curve and model quality. For consistency of measurements, a 20-in LCD computer screen with a resolution of 1680 × 1050 pixels and 0.258 mm diagonal dot pitch with 32-bit color was used. When teeth were severely misaligned, images were rotated on the screen to check that the sectioning line was properly oriented. Magnifying feature was used to enlarge images as needed.

This procedure was performed for all initial (T0) and final (T1) imported STL files for the 75 subjects. The I-IPR per tooth was calculated by the difference in mesiodistal width of each tooth from pre-treatment (T0) to post-treatment (T1) (i.e., the most recent model available). The amount of P-IPR per tooth was calculated by dividing the amount of IPR that is planned for each interproximal area on ClinCheck® by 2, with the assumption that the digitally P-IPR illustrated on ClinCheck® indicates equal amounts reduced on both adjacent teeth (50% on the mesial tooth and 50% on the distal tooth). Therefore, mesial and distal P-IPR for each tooth was calculated and the total P-IPR per tooth was determined. Difference between I-IPR and P-IPR for each tooth was calculated using the following formula:

$${\text{IPR}}\;{\text{difference = }}\left( {\text{P - IPR}} \right) - \left( {\text{I - IPR}} \right)$$

### Statistical analysis

Interrater reliability was assessed by intraclass correlation coefficients. *p*-values smaller than 5% were considered statistically significant.

Sample size calculation (power analysis) was done in a post hoc manner to determine whether the number of subjects included in the study was able to reject our null hypothesis that there is no statistical difference between implemented and planned IPR. Based on this analysis, using the G*power software, a sample of 75 subjects allowed us to detect a 0.07-mm mean difference (very minimal differences) between the implemented and the programmed IPR with 85% power at 5% significance level.

**Fig. 3:**
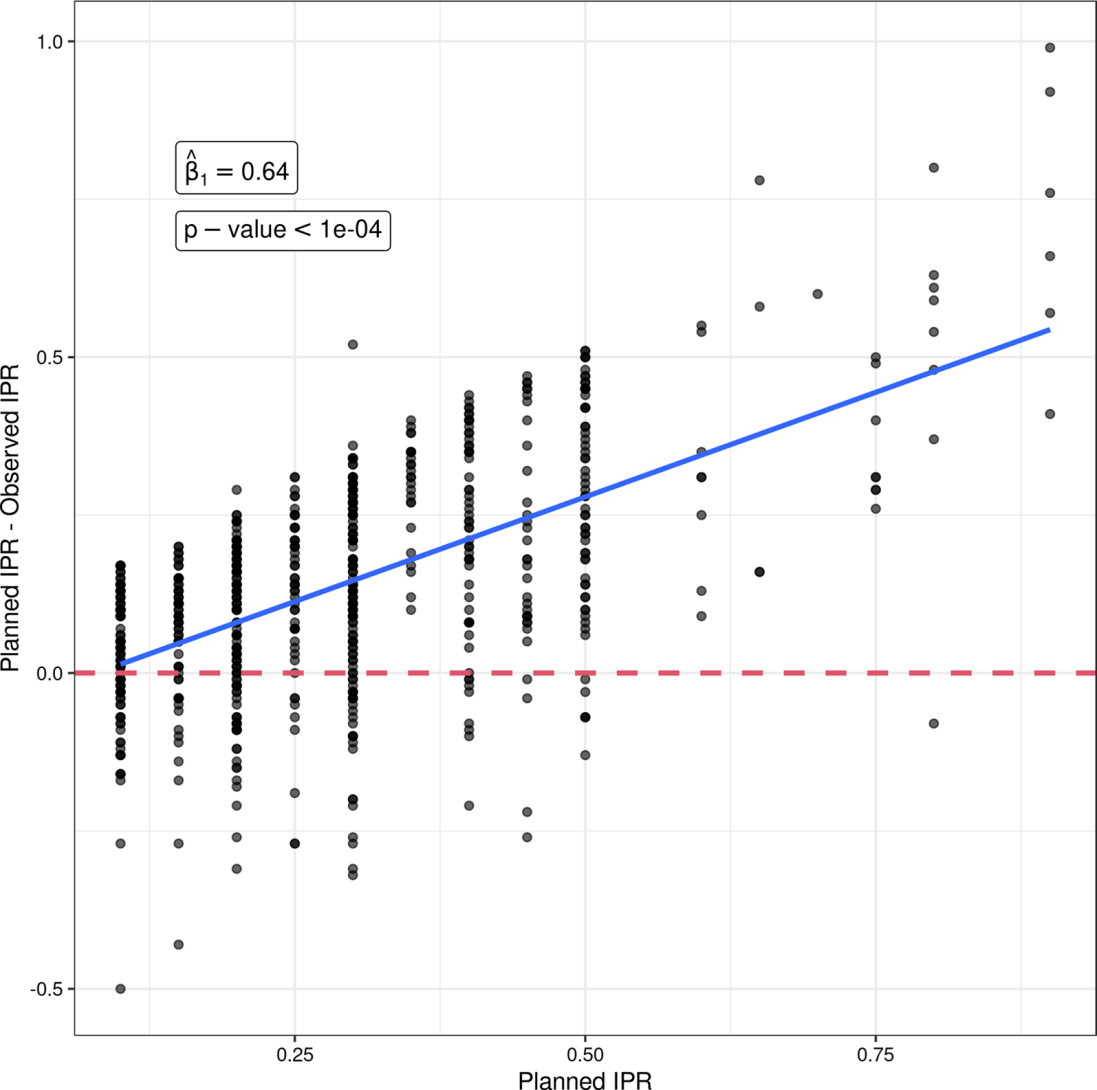
Association between P-IPR to the discrepancy between implemented and programmed IPR

Mean and SD were used for the descriptive statistics. Paired t-tests were used to evaluate differences between i-IPR and p-IPR averaged across teeth within individuals (Table 1). Independent t-tests were used to evaluate tooth group comparisons (Table 2). Groups of teeth were defined as anteriors (incisors and canines), posteriors (premolars), upper (maxillary teeth) and lower (mandibular teeth). P-IPR was associated with difference between P-IPR and I-IPR at tooth level using a linear mixed effects model with a random intercept per subject (Fig. 3). In other words, a linear mixed effects model was fitted to estimate the amount of discrepancy between the P-IPR and the I-IPR per millimeter increase in P-IPR. All the statistical analyses were performed in R version 4.1.0.

**Table 1:** Outcomes for the mean differences between programmed and implemented IPR

Region	Tooth	n teeth	Programmed IPR (P-IPR)	Implemented IPR (I-IPR)	(P-IPR)-(I-IPR)	95% CI for mean IPR difference	*p* value
LOWER	LL5	9	0.22 ± 0.15	0.15 ± 0.16	0.07 ± 0.10	[− 0.008; 0.153]	0.0724
	LL4	34	0.20 ± 0.10	0.11 ± 0.14	0.09 ± 0.16	[0.031; 0.139]	0.0031**
	LL3	45	0.29 ± 0.17	0.14 ± 0.16	0.16 ± 0.20	[0.1; 0.218]	< .0001****
	LL2	46	0.35 ± 0.16	0.20 ± 0.16	0.14 ± 0.20	[0.077; 0.199]	< .0001****
	LL1	47	0.34 ± 0.16	0.17 ± 0.15	0.17 ± 0.18	[0.121; 0.227]	< .0001****
	LR1	47	0.34 ± 0.16	0.20 ± 0.15	0.13 ± 0.16	[0.085; 0.18]	< .0001****
	LR2	47	0.34 ± 0.17	0.18 ± 0.17	0.17 ± 0.22	[0.103; 0.23]	< .0001****
	LR3	42	0.30 ± 0.16	0.14 ± 0.18	0.17 ± 0.23	[0.096; 0.24]	< .0001****
	LR4	31	0.23 ± 0.11	0.14 ± 0.12	0.09 ± 0.15	[0.032; 0.141]	0.0030**
	LR5	14	0.38 ± 0.25	0.25 ± 0.19	0.12 ± 0.18	[0.02; 0.225]	0.0232*
	Overall	362	0.31 ± 0.17	0.17 ± 0.16	0.14 ± 0.19	[0.12; 0.16]	< .0001****
UPPER	UR5	8	0.45 ± 0.21	0.20 ± 0.17	0.25 ± 0.19	[0.095; 0.405]	0.0065**
	UR4	17	0.28 ± 0.13	0.15 ± 0.15	0.13 ± 0.16	[0.05; 0.216]	0.0038**
	UR3	24	0.25 ± 0.14	0.12 ± 0.19	0.12 ± 0.16	[0.051; 0.189]	0.0016**
	UR2	23	0.27 ± 0.16	0.18 ± 0.14	0.09 ± 0.16	[0.025; 0.165]	0.0104*
	UR1	27	0.28 ± 0.16	0.13 ± 0.11	0.16 ± 0.18	[0.086; 0.225]	< .0001****
	UL1	27	0.27 ± 0.17	0.13 ± 0.11	0.13 ± 0.19	[0.056; 0.204]	0.0013**
	UL2	22	0.27 ± 0.16	0.16 ± 0.19	0.11 ± 0.18	[0.028; 0.186]	0.0103*
	UL3	26	0.23 ± 0.13	0.15 ± 0.16	0.08 ± 0.18	[0.008; 0.154]	0.0313*
	UL4	19	0.27 ± 0.14	0.13 ± 0.11	0.14 ± 0.16	[0.061; 0.214]	0.0013**
	UL5	11	0.39 ± 0.22	0.19 ± 0.16	0.20 ± 0.12	[0.119; 0.283]	0.0003***
	Overall	204	0.28 ± 0.16	0.15 ± 0.15	0.13 ± 0.17	[0.105; 0.153]	< .0001****
LOWER + UPPER	Total number of teeth	566	0.30 ± 0.16	0.16 ± 0.16	0.14 ± 0.18	[0.121; 0.151]	< .0001****

*LL5* lower left second premolar, *LL4* Lower left first premolar, *LL3* lower left canine, *LL2* lower left lateral incisor, *LL1* lower left central incisor, *LR1* lower right central incisor, *LR2* lower right lateral incisor, *LR3* lower right canine, *LR4* lower right first premolar, *LR5* lower right second premolar, *UR5* upper right second premolar, *UR4* Upper right first premolar, *UR3* Upper right canine,*UR2* Upper right lateral incisor, *UR1* Upper right central incisor,*UL1* Upper left central incisor, *UL2* Upper left lateral incisor, *UL3* Upper left canine, *UL4* Upper left first premolar, *UL5* Upper left second premolar, *IPR* Interproximal reduction, *P-IPR* Programmed IPR, *I-IPR* Implemented IPR, *CI* Confidence interval

*P < .05; **P < .01; ***P < .001, ****P < .0001

**Table 2:** Between-group comparisons in the mean differences between programmed and implemented IPR

Group	Region	*n* patients	*n* teeth	Programmed IPR (P-IPR)	Implemented IPR (I-IPR)	(P-IPR) − (I-IPR)	95% CI for mean IPR difference	*p*. Value for difference between P-IPR and I-IPR	*p* Value for between group comparison of difference between P-IPR and I-IPR
All (anterior and posterior)	Lower arch	53	362	0.31 ± 0.17	0.17 ± 0.16	0.14 ± 0.19	[0.12; 0.16]	< .0001****	0.4783
	Upper arch	43	204	0.28 ± 0.16	0.15 ± 0.15	0.13 ± 0.17	[0.105; 0.153]	< .0001****	
All (anterior and posterior)	Left side	74	286	0.29 ± 0.16	0.15 ± 0.15	0.13 ± 0.18	[0.112; 0.154]	< .0001****	0.6789
	Right side	71	280	0.30 ± 0.17	0.17 ± 0.16	0.14 ± 0.19	[0.117; 0.161]	< .0001****	
Anterior teeth (1,2,3)	Lower	53	274	0.33 ± 0.16	0.17 ± 0.16	0.16 ± 0.20	[0.133; 0.18]	< .0001****	0.0302*
	Upper	42	149	0.26 ± 0.15	0.14 ± 0.15	0.12 ± 0.17	[0.087; 0.144]	< .0001****	
Anterior teeth (1,2,3)	Left	73	213	0.30 ± 0.16	0.16 ± 0.16	0.14 ± 0.19	[0.114; 0.165]	< .0001****	0.7708
	Right	70	210	0.30 ± 0.16	0.16 ± 0.16	0.14 ± 0.19	[0.119; 0.171]	< .0001****	
Posterior teeth (4,5)	Lower	38	88	0.24 ± 0.15	0.15 ± 0.15	0.09 ± 0.15	[0.058; 0.122]	< .0001****	0.0059**
	Upper	22	55	0.32 ± 0.18	0.16 ± 0.14	0.17 ± 0.16	[0.122; 0.208]	< .0001****	
Posterior teeth (4,5)	Left	48	73	0.25 ± 0.15	0.13 ± 0.14	0.11 ± 0.15	[0.08; 0.15]	< .0001****	0.7413
	Right	44	70	0.29 ± 0.17	0.17 ± 0.15	0.12 ± 0.17	[0.084; 0.163]	< .0001****	

*P < .05; **P < .01; ***P < .001, ****P< .0001

## Results

### Reliability assessment

An overall good reliability was reported between the examiners for teeth from a random sample of 14 patients (intraclass correlation coefficient 0.81, 95% CI: 0.76 to 0.85), (“Appendix”).

### Main findings

Assessment was carried on STL files for 75 subjects, and 43 had IPR prescribed in the upper arch only; 53 patients had IPR prescribed in the lower arch, and 21 patients had IPR prescribed in both upper and lower arches. The teeth prescribed for IPR varied and only included those between second premolar to second premolar on the opposite side for both maxillary and mandibular arches. A total of 362 lower arch teeth and 204 upper arch teeth had IPR prescriptions and consequently were analyzed (Fig. 1).

Statistically significant differences were observed between the overall average value of digitally programmed and implemented IPR per tooth for both the maxillary (*p* < 0.0001) and mandibular (*p* < 0.0001) teeth (second premolar to second premolar). The mean P-IPR for the maxillary teeth was 0.28 ± 0.16 mm versus the mean I-IPR 0.15 ± 0.15 mm. Likewise, in the mandibular arch, the mean P-IPR for mandibular teeth was 0.31 ± 0.17 mm, while it was 0.17 ± 0.16 mm for the implemented. The mean I-IPR was consistently lower than the mean P-IPR regardless of teeth and sites (*p* < 0.0001, Tables 1 and 2). The discrepancy was more evident for the lower anterior teeth than for the upper anterior teeth (*p* = 0.0302) and more for the upper posterior teeth compared to the lower posterior teeth (*p* = 0.0059), as given in Table 2.

Figure 3 illustrates the difference between the programmed and implemented IPR in relation to P-IPR. Increased P-IPR was associated with increased discrepancy between P-IPR and I-IPR. Mean discrepancy increases by 0.64 mm (*p* < 0.0001) per unit (mm) increase in P-IPR.

## Discussion

Evaluation of the consistency between the pre-planned ClinCheck® IPR, which is often adjusted by the clinician according to the clinical case requirement, and that implemented clinically has received very little scientific evaluation, especially for teeth in each quadrant. A very careful space analysis is required prior to any IPR-based treatment; additionally, the accurate implementation of the desired IPR is likely essential to achieving desired goals and treatment objectives. The evaluation of 75 STL ClinCheck® initial and final models has revealed that IPR was often carried out in the lower arch with a total of 362/566 (64%) teeth being slenderized to resolve lower crowding compared to 204/566 (36%) teeth slenderized in the upper arch. This agrees with previous findings that assessed Invisalign® treatment results and found that 58% and 48% of mandibular and maxillary crowding, respectively, were resolved by IPR [18]. Furthermore, our sample primarily included adult patients, aged 38 ± 15 years on average; therefore, it is also expected to see a more crowded lower dentition than on the upper arch, attributed to what is known as late incisors’ crowding, which is exhibited in individuals over 20 years of age [19] and considered to be multifactorial [20, 21].

Data assembled from the total of 566 upper and lower teeth have shown that the overall average amount of I-IPR for the upper and lower teeth was significantly smaller than that programmed in the ClinCheck®, even when teeth were analyzed individually, except for the lower left second premolar (Table 1). Two previous studies reported reduced amount of the I-IPR in clear aligner therapy compared to that initially programmed in the ClinCheck®[14, 16]. De Felice et al. [14] investigated differences between planned and performed IPR in 25 cases (total arch measurements) and found that the difference was on average 0.55 ± 0.64 mm (*p* < 0.05) in the upper arch and 0.82 ± 0.84 mm (*p* < 0.05) in the lower arch; they had an accuracy of 44.95% for the IPR in the upper and 37.02% for the lower arch. In our study, using their same accuracy formula, we found approximately 45% and 46% accuracy in the mandible and maxilla, respectively. Our study is the first to estimate the difference between the average P-IPR vs I-IPR per tooth (right and left) in both upper and lower arches and compare this difference between similar teeth in different regions (Table 2). It revealed a statistically highly significant difference between the average P-IPR and I-IPR per tooth for the lower (0.14 ± 0.19 mm, *p* < 0.0001) and upper (0.13 ± 0.17 mm, *p* < 0.0001) teeth. This may not only indicate a statistically significant level but such a high level of inadequate I-IPR might be considered clinically significant, especially considering that when we analyzed teeth individually the statistically significant differences were evident for almost all teeth (Table 1).

Similar findings to our study were reported by another observational study [16]. In that study, the overall difference between implemented and programmed IPR for 464 teeth was on average 0.15 ± 0.14 mm (*p* = 0.0001) compared to our findings for 566 teeth (0.14 ± 0.18 mm, *p* < 0.0001) with the implemented IPR per tooth being less than that digitally programmed. This study had a smaller sample size and did not evaluate the average IPR difference per tooth for each quadrant (right or left) but rather grouped the teeth into incisors, canines and premolars. On the other hand, our findings disagree with a recent study by Lagana et al. [17] whom reported that the amount of IPR performed in vivo correlates with that planned by the orthodontists in ClinCheck®. They retrospectively studied digital models for 30 subjects and measured the widest mesiodistal diameter for each tooth pre- and post-treatment using the OrthoCAD® software and calculated the average P-IPR vs I-IPR for the upper and lower arches. In this study, each tooth was 2D sectioned from its mesial to its distal height of contour using the Ortho Analyzer software cross-sectional tool; this helped improve the accuracy of visualizing the maximum width of the teeth between the mesial and distal maximum contact point contours. Therefore, the different measurement tools used for assessing the amount of IPR and the smaller sample size in their study might have attributed to these differences in outcomes.

The precision of implementing the planned IPR clinically might be possibly related to three factors: technical-, operator- and patient-related factors. Manual and mechanical techniques (technical factors) are usually undertaken in clinical orthodontics for precise IPR implementation: the traditional hand pulled strips, oscillating segmented disks and motor-driven abrasive strips. Accuracy of these procedures has been previously investigated; the results seem to be controversial [16, 22, 23]. One study reported that upon quantitative evaluation of the stripped enamel between these three commonly used stripping procedures, great variability was noticed, with all of these techniques delivering less IPR than intended [23]. On the other hand, Danish et al. [22] reported smaller amount of removed enamel using Ortho-Strips, compared to metal strip and air rotor IPR. In our study, operators used a combination of all these techniques for slenderizing, which might have contributed to having an overall average I-IPR per tooth less than what is programmed in the ClinCheck®.

As for operator-related factors, orthodontists are often conservative in initiating the stripping process. And while performing IPR, minimal enamel amounts are often slenderized symmetrically from the prescribed contact areas before the maximal (planned) amount is reached. To avoid over-reduction and side effects related to sensitivity and pulpal irritation, especially in narrow and crowded teeth, clinicians subconsciously reduce conservative amounts and less than what is prescribed. This is often seen if the amount of crowding is significant, where the amount of programmed IPR is increased, which makes it challenging to break the contact between teeth and perform symmetrical IPR. Therefore, in such cases, clinicians might reduce less amount of enamel than what is programmed. This aligns with the finding that the mean P-IPR is consistently greater than the mean I-IPR regardless of P-IPR, and the mean discrepancy increases by 0.64 mm (*p* < 0.0001) per unit (mm) increase in P-IPR (Fig. 3).

As for patient-related factors, there is also a possibility that the location, anatomy, periodontal condition of the teeth play a role in the precision of implementing the planned IPR, especially considering that interproximal enamel thickness varies among individuals and teeth [24, 25]. Therefore, our study also aimed to assess the correspondence between digitally planned and implemented IPR for individual teeth (second premolar to second premolar) in the upper and lower arches. Most of the teeth displayed a statistically significant difference between the amount of the programmed and implemented IPR. In the lower arch, the highest discrepancy was exhibited for all anterior teeth (canine to canine, *p* < 0.0001) with a tendency toward inadequate amount of I-IPR (Table 1). Greater precision was observed for the lower premolars, with the lower left second premolar (LL5) showing the greatest correspondence between the planned and implemented IPR (*p* = 0.0724). It can be assumed that the majority of clinicians performing IPR in this study were right-handed whom had better access to the left quadrant of the jaw than the right. It was also noticed that P- IPR for the lower anterior teeth (0.33 ± 0.16 mm) is on average greater than that prescribed for the lower posteriors (0.24 ± 0.15 mm) and that prescribed for the upper anteriors (0.26 ± 0.15 mm, Table 2). This is probably due to the greater amount of crowding often encountered in the lower anterior region, especially for adults [26]. On the other hand, more P-IPR was prescribed for the upper posterior teeth (0.32 ± 0.18 mm) compared to lower posteriors (0.24 ± 0.15 mm). This is possibly due to the need for provision of posterior spacing to achieve Class I canine relationship in Class II malocclusion cases [27]. Moreover, our results indicated that IPR difference (P-IPR-I-IPR) was more evident for lower anterior teeth than for upper anterior teeth (*p* = 0.0302) and on the contrary had a larger discrepancy in the upper posterior teeth than lower posterior teeth (*p* = 0.0059) (Table 2). This can be explained by the greater prescribed P-IPR in both mandibular anterior and maxillary posterior regions (Table 2). Another explanation may be related to the accessibility of the interproximal surface to reduce. The mandibular anterior region is often more crowded than any other jaw segment, especially in adults [20], resulting in tipping, distortions, and very tight interproximal contacts between these teeth, which eventually hinder the smoothness of performing IPR in this region. Accessibility of the upper posterior segments compared to the lower posterior segments is more challenging for the clinician to control the IPR procedure while using indirect visualization technique. This premise is supported by Kalemaj et al. [16] who reported lesser discrepancy between planned and implemented IPR for lower premolars and higher discrepancy for the mandibular canines. Finally, this observed imprecision might be associated with the stretching of the periodontal ligament while performing IPR and using the measuring gauge in a crowded area, that it might falsely appear to the clinician that the desired amount of enamel reduction has been achieved [28, 29].

Even though ClinCheck ®offers an IPR timing that is automatically staged when access to interproximal contacts is feasible [30]. The amount of IPR performed clinically even in clear aligner therapy is still under the influence of enamel hardness, tooth morphology, pressure applied, size of the abrasive tool and polishing procedures [31]. As mentioned previously, technical-, operator- and patient (teeth)-related factors play a role in the accuracy of implementing the planned IPR for any desired treatment plan. Therefore, precision in implementing the digitally programmed IPR remains challenging, and clinicians should pay extra attention to attain the planned IPR as requested. Otherwise, the tracking of the aligners could be compromised, and case refinement ClinCheck® may be required. Increasing the precision of the IPR can be achieved by using the predetermined thickness disks and the measuring gauges, the progressive reduction of enamel and the use of wedges for teeth separation [32].

Finally, the cross-sectional technique used for measurements in this study proved to be superior to what has been previously utilized to assess IPR; it provided an advantage over measuring the mesiodistal teeth from contact point to contact point on digital or plaster casts. Each tooth was individually isolated and cross-sectioned along its long axis; this allowed accurate measurement for the data. The accuracy of the calibrated digital model measuring tool of the Ortho Analyzer software has been investigated, and its reliability and precision were confirmed [33]. More recently, intraoral direct measurements taken in individuals’ oral cavity with a 0.01-mm accuracy digital caliper were compared to measurements using the 3 Shape Ortho Analyzer cross-sectional tool; results indicated that these measurements are accurate replica and as reliable as direct measurements [34].

### Limitations

The main limitation of this study is that it was a retrospective evaluation for STL models from ClinCheck® for patients treated or undergoing treatment with clear aligners. Multiple potential confounding factors were present due to patients being treated by several providers with various clinical experiences who utilized different IPR procedures. Intrarater reliability was not assessed, since the accuracy and reliability of the Ortho Analyzer software have been studied, and its calibrated digital measuring tool has proved to be accurate as indicated previously [33].

## Conclusion

The amount of implemented-IPR in clear aligner therapy seems to be less than that digitally programmed for most teeth. Regardless of the region, I-IPR was consistently lower than that programmed. Mandibular anterior teeth and maxillary posterior teeth showed greater discrepancy between P-IPR and I-IPR, than the maxillary anterior and mandibular posterior teeth. Further prospective studies should be undertaken to determine the factors affecting the precision of implementing IPR clinically and the clinical implications of a significantly reduced I-IPR on the accuracy of clear aligner’s treatment outcomes.

### Implications for research and clinical practice

IPR might be one of many factors that may influence the effectiveness of clear aligner therapy and the need for refinements. The focus of this research was primarily to assess whether the difference between the amount of implemented to that of programmed IPR truly exists. It has been shown that the overall amount of implemented IPR seems to be significantly less than what the treatment plan requires; therefore, the clinical implications of this discrepancy on the overall occlusal results of treatment need to be determined, and future research should uncover the effect of this discrepancy on the treatment outcomes and need of refinements in clear aligner therapy.

## Data Availability

The data underlying this article are available in the article and its online material.

## Appendix

See Table 3.

**Table 3:** Intraclass correlation coefficient to assess interexaminer reliability

Tooth	ICC	Lower bound	Upper bound
LL5	0.68	− 0.583	0.998
LL4	0.68	− 0.001	0.940
LL3	0.90	0.746	0.970
LL2	0.90	0.715	0.971
LL1	0.87	0.633	0.960
LR1	0.80	0.493	0.934
LR2	0.92	0.767	0.973
LR3	0.79	0.476	0.928
LR4	0.80	0.335	0.955
LR5	0.73	− 0.019	0.966
UR5	0.06	− 0.9772	0.990
UR4	0.68	− 0.197	0.980
UR3	0.62	0.115	0.880
UR2	0.13	− 0.178	0.587
UR1	0.73	0.193	0.937
UL1	0.47	− 0.230	0.866
UL2	0.84	0.376	0.972
UL3	0.94	0.796	0.986
UL4	0.51	− 0.151	0.959
UL5	0.75	− 0.098	0.985

## Abbreviations

I-IPR: Implemented IPR
IPR: Interproximal reduction
LL5: Lower left second premolar
P-IPR: Programmed IPR
STL: Stereolithography models
UR2: Upper right lateral Incisor
UR3: Upper right canine
UR4: Upper right first premolar
UR5: Upper right second premolar
